# Specimen, biological structure, and spatial ontologies in support of a Human Reference Atlas

**DOI:** 10.1038/s41597-023-01993-8

**Published:** 2023-03-27

**Authors:** Bruce W. Herr, Josef Hardi, Ellen M. Quardokus, Andreas Bueckle, Lu Chen, Fusheng Wang, Anita R. Caron, David Osumi-Sutherland, Mark A. Musen, Katy Börner

**Affiliations:** 1 Department of Intelligent Systems Engineering, Luddy School of Informatics, Computing, and Engineering, Indiana University, Bloomington, IN 47408 USA; 2 Stanford Center for Biomedical Informatics Research, Stanford University, Stanford, CA 94305 USA; 3 Department of Computer Science, Stony Brook University, Stony Brook, NY 11794 USA; 4 Department of Biomedical Informatics, Stony Brook University, Stony Brook, NY 11794 USA; 5 European Bioinformatics Institute (EMBL-EBI), Wellcome Genome Campus, Hinxton, UK

**Keywords:** Classification and taxonomy, Software

## Abstract

The Human Reference Atlas (HRA) is defined as a comprehensive, three-dimensional (3D) atlas of all the cells in the healthy human body. It is compiled by an international team of experts who develop standard terminologies that they link to 3D reference objects, describing anatomical structures. The third HRA release (v1.2) covers spatial reference data and ontology annotations for 26 organs. Experts access the HRA annotations via spreadsheets and view reference object models in 3D editing tools. This paper introduces the Common Coordinate Framework (CCF) Ontology v2.0.1 that interlinks specimen, biological structure, and spatial data, together with the CCF API that makes the HRA programmatically accessible and interoperable with Linked Open Data (LOD). We detail how real-world user needs and experimental data guide CCF Ontology design and implementation, present CCF Ontology classes and properties together with exemplary usage, and report on validation methods. The CCF Ontology graph database and API are used in the HuBMAP portal, HRA Organ Gallery, and other applications that support data queries across multiple, heterogeneous sources.

## Introduction

The Human BioMolecular Atlas Program (HuBMAP)^1^ is developing a human reference atlas (HRA) of the trillions of cells in the human body^2^, in close collaboration with 16 other consortia^3^. The HRA aims to serve the needs of biomedical researchers, practitioners, teachers, and learners. It attempts to capture healthy human diversity (e.g., the 3D location and types of cells and associated biomolecular data), and it will be useful to biomedical experts for many purposes. One major use case is allowing researchers to assess changes in the numbers and types of cells due to aging and disease.

## Human Reference Atlas Construction and Usage

The third HRA (v1.2) was released in June 2022, and it includes 26 organ-specific Anatomical Structures, Cell Types plus Biomarkers (ASCT+B) tables and 54 associated 3D reference organ models, among other features. On August 25, 2022, the HRA had been successfully used across four consortia to spatially register 350 tissue blocks into the HRA common coordinate framework. On December 12, 2022, the count had increased to 1,161 tissue blocks, and these tissue blocks link to more than 6,000 biomolecular datasets derived from these tissue samples. In June 2022, the CCF Ontology was published for the first time via BioPortal (https://bioportal.bioontology.org/ontologies/CCF, v2.0.1) and the EBI Ontology Lookup Service (OLS) Ontology Browser (https://www.ebi.ac.uk/ols/ontologies/ccf, v2.0.1) in order to support programmatic access to data elements needed for HRA construction and usage.

### Human Reference Atlas

To construct the HRA, it is necessary to standardize experimental specimen metadata and to have a common coordinate framework (CCF, https://commonfund.nih.gov/sites/default/files/CCFsummaryfinal.pdf) that provides a biological structure vocabulary linked to 3D spatial data for every organ, tissue, and cell in the human body^3^. To construct the CCF for the HRA, we assume that (1) anatomical structures are composed of cells that have different types (i.e., cell types are located in anatomical structures), combinations of biomarkers characterize different cell types, and experts are interested in querying for those entities (i.e., we need standardized terminology to query for anatomical structures, cell types, and biomarkers). We call this *biological structure information*. (2) The 3D space in which anatomical structures, cells of different types, and biomarkers operate matters. We call this *spatial information*. (3) Human sex, age, and genetic factors effect the structure and function of anatomical structures, cells, and biomarkers. We call this *specimen information*, and we use it to capture human diversity and its impact on body structures and functions.

### Biological structure information

**Fig. 1:**
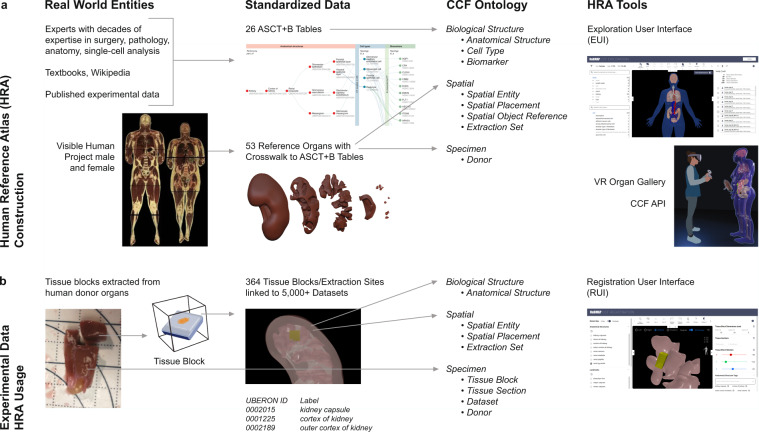
From Real-World Entities via Standardized Data to CCF Ontology used by HRA Tools. (**a**) Human Reference Atlas construction takes real-world data and represents it in standardized data structures that are defined by the interlinked Biological Structure, Spatial, and Specimen data in the CCF Ontology. Anatomical structures in the ASCT+B tables are linked via *part_of* relationships resulting in a partonomy; they are crosswalked to 3D reference organs in support of spatial queries and exploration using different HRA tools shown on the right. (**b**) HRA usage includes search for specific cell types or biomarker expression values across organs in a 3D reference space. Users can also upload new experimental data (e.g., a new tissue block from a human donor specimen); the tissue is registered into the HRA by spatially mapping it to a 3D reference organ. If the Registration User Interface (RUI) shown on left is used, anatomical structure tags (see UBERON ID and Label) are automatically assigned based on collision events; spatial search becomes possible in the Exploration User Interface (EUI); and cell types or biomarkers associated with the colliding anatomical structures can be retrieved via ASCT+B tables and explored in the EUI.

Anatomical structures, cell types, and biomarkers (ASCT+B) tables record the biomarker sets that characterize cell types within the nested anatomical structures that compose an organ. The ASCT+B tables serve as metadata templates for community standards^4^, making data more findable, accessible, interoperable, and reusable (FAIR)^5^. The ASCT+B tables are authored by human experts using templated Google Sheets—see standard operating procedure (SOP) entitled “SOP: Authoring ASCT+B Tables”^6^. The biomarkers, cell types, and anatomical structures are mapped to existing ontologies if they are available (e.g., to Uberon^7,8^). An exemplary ASCT+B table with an anatomical structure partonomy (in red on left) linked both to cell types (in blue in middle) and biomarkers (in green on right) is shown in Fig. 1a. All existing ASCT+B tables can be interactively explored using the ASCT+B Reporter (https://hubmapconsortium.github.io/ccf-asct-reporter).

### Spatial information

The 3D Reference Object Library captures the shape, size, location, and rotation of major anatomical structures. They are authored by medical illustrators following the “SOP: 3D Reference Object Approval”^9^. For each organ, there exists a digital representation of the so-called scene graph that represents all 3D objects (i.e., anatomical structures) and inter-object relationships (e.g., what object is inside or next to which other object). There exists a crosswalk table that associates 3D object names with the anatomical structure terms in the ASCT+B tables that link to terminology in existing ontologies whenever possible. Figure 1a depicts how Visible Human Project data (VHP)^10^ (a high-quality, whole-body dataset published by the National Library of Medicine) is used to create reference organs (different anatomical structures of a kidney are shown) and how resulting data is uniformly represented using the spatial and specimen information. All reference organs can be interactively examined in the Exploration User Interface (EUI, https://github.com/hubmapconsortium/ccf-ui) and the HRA Organ Gallery in virtual reality (VR)^11^, both of which are detailed in the CCF Ontology Applications section. The spatial size, shape, location, and rotation of experimental tissue data is captured relative to the organs in the 3D Reference Object Library. Uniform tissue registration is facilitated by the Registration User Interface (RUI) (Fig. 1b, **right**).

**Fig. 2:**
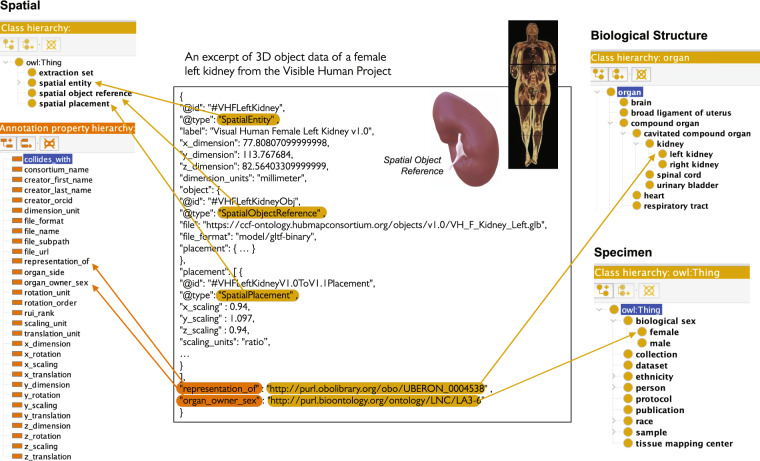
HRA reference organ description. The Visible Human Project female left kidney (#VHFLeftKidney) reference organ is represented as a *Spatial Entity* class of type *Spatial Object Reference*, and with a *Spatial Placement*. Properties such as *representation_of* with PURL link to UBERON:0004538 (left kidney) refer to Biological Structure data, whereas properties such as *organ_owner_sex* with PURL link to LNC:LA3–6 (female) refer to Specimen data.

### Specimen information

Data capturing the sex, age, and other information on donors that provided tissue data used in the construction of the HRA are tracked. For the HRA (v1.2), data from the Visible Human Project was used in the construction of the 3D reference organs—the male was white, 180.3 cm (71 inch) tall, 90 kg (199 pound) and was 38 years old; the female was white, 171.2 cm (67.4 inch) tall, obese (weight unknown), and 59 years old.

### Scholarly and Experimental Evidence

We track scholarly publications^42^ and experimental studies that provide evidence for entities in the HRA (e.g., for what cell types have what gene expressions in which tissues). In addition, we capture data-processing provenance so the HRA can be recomputed, as needed, when new data and algorithms become available.

### HRA construction versus usage

Just as with geospatial maps, there is a difference between atlas creation (i.e., defining the reference space) and atlas usage (e.g., finding a place in the reference space or a pathway between two locations). Figure 1 provides an overview of HRA construction and usage by experts (e.g., anatomists, pathologists, surgeons, single cell researchers). We show real-world entities on the left, standardized data in the middle, and CCF ontologies and value-added services on the right. Figure 1a shows HRA construction that includes the creation, validation, and publishing of the CCF ontologies and associated APIs and tools. It starts with the creation of ASCT+B tables by experts with decades of expertise in surgery, pathology, anatomy, single-cell analysis, who consult textbooks, Wikipedia, and published experimental data. In parallel, professional medical illustrators model 3D spatial reference organs in support of tissue registration (e.g., the resulting reference organs have anatomical structures that serve as landmarks when registering virtual tissue blocks and can be used to assign semantic tags). Last but not least, crosswalks are compiled that link all 3D reference objects to anatomical structures in the ASCT+B tables (see details in Börner *et al*., 2021^3^). Figure 1b details HRA usage—e.g., the mapping of new experimental tissue data into the HRA so it can be compared across tissue donors, laboratories, assay types. Alternatively, the HRA can be used to query for cell types and the genes they express in different locations of the 3D human body. We show how tissue blocks are cut from human organs (and possibly further cut into tissue sections). The tissue blocks are then spatially registered into 3D reference organs using the Registration User Interface (https://hubmapconsortium.github.io/ccf-ui/rui/), shown on right (and discussed in the CCF Ontology Applications section), that assigns anatomical structure annotations (Uberon IDs and labels) via 3D object collision detection (see papers^12^ and the CCF Ontology Applications section); Assigned anatomical structures and data in the ASCT+B tables are then used to predict cell types and gene expressions for a tissue block. HRA-predicted data can then be compared to experimental data and the HRA data is revised as needed to capture healthy human diversity. Note that only the highest quality experimental data is used for HRA construction; all CCF-registered data can be explored within the HRA.

### Common coordinate framework ontology

The CCF Ontology detailed in this paper formalizes and interlinks major classes that describe biological structure, spatial, and specimen information captured in the HRA. It thus provides a unifying vocabulary for HRA construction and usage—making it possible to ingest external data sources; supporting uniform tissue sample registration that includes the spatial positioning and semantic annotations within the 3D reference organs; and supporting user-formulated cross-domain queries over tissue donor properties, anatomical structures, cell types, biomarkers, and 3D space. The use of the CCF Ontology as a framework for the HRA makes it possible to publish HRA data in the Linked Open Data (LOD) cloud^13^ in a Findable, Accessible, Interoperable and Reusable (FAIR)^5^ manner; the CCF API supports programmatic access to HRA data. We provide templates via data specifications so that other consortia can easily publish their data in a HRA-compatible manner.

## User Needs That Guide CCF Ontology Modeling

There is a major difference between (1) the *construction* of the HRA and its underlying common coordinate framework (i.e., developing a latitude/longitude-like system) and agreeing on a way to map new data onto it and (2) the *usage* of the HRA to run different semantic and spatial queries. It is critically important to understand the goals and user needs for both parts to guide CCF Ontology design and HRA tool development (see examples in the CCF Ontology Applications section). At their core, anticipated HRA queries act as competency questions^14^ that inform the modeling of the CCF Ontology.

### HRA construction

Both—human expertise and the highest quality experimental data—are used to construct the HRA. Some data exist only at the gross anatomy level (e.g., data covered in anatomy books or Visible Human Project data), whereas other data captures single-cell observations involving micro-anatomical structures (e.g., gene expression data for cells). All data—not only human expertise but also experimental data across different levels of resolution—must be registered into the very same HRA CCF. To register experimental data (currently ca. 6,000 datasets) collected from thousands of human donors (currently ca. 200), it must be uniformly registered and annotated in terms of biological structure, spatial, and specimen characteristics. HRA terms should link to existing ontologies whenever possible. If HRA terms do not exist in reference ontologies, then a GitHub issue is submitted to the respective ontology developers for eventual inclusion of these anatomical structures and cell types into existing ontologies, and such requests are tracked (https://github.com/obophenotype/uberon, https://github.com/obophenotype/cell-ontology). Collision detection at the surface mesh level using the 3D Geometry-Based Tissue Block Annotation tool (https://github.com/hubmapconsortium/ccf-tissue-block-annotation, see CCF Ontology Applications section) needs to be run to identify what type and volume of anatomical structures a tissue block contains (this step is necessary as most tissue samples cover cells from multiple anatomical structures). Datasets for these tissue blocks (e.g., cell type name annotations computed using tools such as Azimuth^15^ or protein expression values measured for cells in experiments) must be mapped to anatomical structures—so it becomes possible to show cell-type populations (i.e., the number of cells per type within an anatomical structure) inside of each 3D anatomical structure. For each HRA entity, all the data and code needed to recompile it must be tracked to ensure reproducibility. A new HRA is computed over the course of several days using massive storage and computing resources every six months. Upon each successful recompilation, baseline HRA statistics such as entity and relationship counts, cell-type populations, or query-performance test results are computed and published.

### HRA usage

Some users are interested in understanding in which 3D spatial locations or in which anatomical structures cells of a certain type (e.g., immune cells) can be found or what biomarkers are available or common for a certain cell type (e.g., are there differences in gene expression values for a certain cell type across organs). Other users would like to compare their very own experimental data to the growing inventory of HRA-compatible data (e.g., to understand how diseased cell-type populations or biomarker-expression values differ from a healthy reference). A third set of users is interested in transferring cell-type annotations from the HRA to a new experimental dataset (e.g., using the Azimuth^15^ tool that automates the analysis and interpretation of single-cell RNA-seq experiment data, and transfers cell annotations from a reference map to new experimental data). If new data is uploaded, it needs to be registered and analyzed at scale; 3D exploration requires that 3D reference organ files are loaded and spatial and other queries are run efficiently using specimen, biological structure, and/or spatial metadata. For example, a user might like to see all tissue blocks for 50-year-old females or search for cell types in a specific spatial location that have high gene-expression values in experimental datasets. The interlinkage of spatial reference objects and ASCT+B tables makes it possible to associate cell-type and biomarker data from experimental data to 3D anatomical structure data and to compute how many cells of what type are commonly found in which anatomical structures and what their typical biomarker expression values are. As the number of HRA-registered datasets increases, prediction accuracy will increase. Because the HRA is spatially explicit, the spatial location of cell-type populations can be visually presented in 3D. Initially, cells are placed randomly; as more data regarding cell-neighborhoods becomes available^16^, cells can be placed in spatially explicit patterns. Usage statistics must be captured to identify bottlenecks (e.g., long loading or computing times) and to improve application performance.

Note that the HRA is continuously evolving as new, higher-quality datasets become available for an increasing number of organs. HRA construction and optimization for different use cases will likely take decades. As with any atlas, the availability of the HRA will inspire qualitatively novel questions and queries that are hard to envision today.

## Results

The CCF Ontology v2.0.1 focuses on the subset of the third HRA (v1.2) release that is used for tissue registration and exploration in the HuBMAP (https://portal.hubmapconsortium.org/) and GTEx data portals (https://gtexportal.org/home/eui) and in other tools and services (see the CCF Ontology Applications section).

### CCF Ontology modeling assumptions and goals

The CCF Ontology is modeled to serve the needs of HRA construction and usage. It formally defines the three interlinked parts of the HRA via three interlinked ontologies, representing (1) biological structure, (2) spatial, and (3) specimen data (see Fig. 1). Here, we discuss CCF Ontology modeling assumptions, goals, and desirable properties.

#### FAIR and interoperable

We want the HRA content to be findable and interoperable with other high-quality data sources outside the HRA. Therefore, HRA terms should be linked, whenever possible, to existing, widely used ontologies. For example, HRA anatomical structures are linked to Uberon^7,8^ (and FMA^17–19^ as needed), and cell types are linked to the Cell Ontology^20,21^. These two ontologies are used extensively to annotate single-cell transcriptomics data (https://cellxgene.cziscience.com/) and are cross integrated with each other and with widely used ontologies (e.g., the Gene Ontology [GO]^22^, the Human Phenotype Ontology^23^, and the Mondo Disease Ontology^24^). HRA gene and protein biomarkers are linked to HUGO Gene Nomenclature Committee (HGNC, https://www.genenames.org/) gene symbols and crosswalks exist to Ensembl^25^ for genes and to UniProt^26^ for proteins.

#### Limitations of existing ontologies

Uberon and CL are overly complex for the needs of the HRA, as they cover multiple species and developmental stages. At the same time, both ontologies lack some terms needed by the HRA. FMA can be hard to query due to inconsistent application of ontology design patterns, and it does not link to other ontologies. Existing reference ontologies for biological structures cannot represent the spatial size, location, and rotation of 3D human reference organs and experimental data relative to the HRA and to each other; some ontologies, such as Uberon and FMA, have incorporated anatomical location descriptors using spatial properties (e.g., *anterior_to, posterior_to, left_of, right_of, opposite_to*, etc.), but these are often too general (e.g., in Uberon the location descriptors are for multiple species).

#### Use existing standards

CCF Ontology defines a common vocabulary to annotate data in an HRA-compliant manner using several well-known biomedical ontologies, including Uberon, FMA, CL, HGNC, and other semantic web vocabularies, such as the Dublin Core™ Metadata Initiative Terms (DCTERMS, http://www.dublincore.org). The CCF Ontology uses some Dublin Core properties for providing provenance information, such as the data publisher, creator, and creation date. The CCF Ontology uses terms from resources in the Open Biological and Biomedical Ontology (OBO) library to define anatomical structures, cell types, and biomarkers. The CCF Ontology is published using the standard Semantic Web Ontology Language (OWL), which is built on the Resource Description Framework (RDF).

#### Validation

The anatomical structures and cell types in the ASCT+B tables are validated against reference ontologies on a weekly basis (see Methods section). Validation results are used to improve the ASCT+B tables and to issue ontology change requests to slowly and steadily modify existing reference ontologies (e.g., Uberon and CL) so that they accurately represent needed information about healthy human adults. When completed, the CCF Ontology will be a proper, HRA-labeled component of the library of major reference ontologies that are widely used by a diverse range of bioinformatics applications.

#### HRA will evolve over time

As new experimental datasets and analysis results become available, the HRA data structures and the CCF Ontology will need to be updated to capture new entity types and linkages and to serve qualitatively new user needs.

### CCF Ontology classes and properties

The CCF Ontology, published as CCF.OWL v2.0.1 (https://bioportal.bioontology.org/ontologies/CCF), comprises three interlinked ontologies that capture information regarding Biological Structure, Spatial relationships, and Specimen characteristics (see Fig. 1). The names of the three ontologies, their classes, properties, and definitions can be found in **Zenodo Table 1**^27^. Whereas the HRA is a large graph-based repository of both data and knowledge, all *descriptions* of the data and all knowledge elements are built from the standardized terms in the CCF Ontology. The ontology thus provides a uniform vocabulary for talking about the large set of digital objects that the HRA encodes.

#### The biological structure information defines three classes

***Anatomical Structure***, ***Cell Type***, and ***Biomarker***. The three classes are derived from the ASCT+B tables for HRA construction. During HRA usage, *Anatomical Structure* annotations are assigned to tissue blocks based on collision events (commonly used in computer games to compute spatial relationships among moving objects, see explanation of mesh-level collision detection in CCF Ontology Applications). In the ASCT+B tables, *Anatomical Structure* terms are linked to Uberon (supplemented with FMA^17–19^ as needed). *Cell Type* terms link mainly to CL^20,21^ (supplemented with Provisional Cell Ontology [PCL] for brain and Human Lung Maturation [LungMAP Human Anatomy; LMHA] for lung, https://bioportal.bioontology.org/ontologies/LUNGMAP-HUMAN). Note that Uberon and CL are already widely used by the single-cell research community, making them an obvious choice. *Biomarker* terms are linked to the HGNC (https://www.genenames.org) standard pipeline developed by the brain data standards initiative, which is used to link these terms to cell type terms.

#### The spatial information defines four classes

***Spatial Entity***, ***Spatial Placement***, ***Spatial Object*** ***Reference***, and ***Extraction Set***. HRA Construction uses all four classes, while HRA Usage uses the first two only. Terms for anatomical structures from the Biological Structure information are linked to 3D reference data in support of spatial registration and spatial exploration. Concretely, each object in a 3D organ is annotated with the appropriate anatomical structure term from the Biological Structure information﻿. Lastly, each instance of a 3D organ has a set of spatial parameters that state its position, size, and orientation in relation to the HRA coordinate space.

#### The specimen information defines four classes

***Tissue Block***, ***Tissue Section***, ***Dataset***, and ***Donor***. Only *Donor* is used for HRA construction; all four are used for HRA usage. For HRA construction, we track Visible Human Project specimen data. For HRA usage, we record *Tissue Block, Tissue Section, Dataset, Donor* info for each tissue block. In terms of *Donor* data that is collected by independent laboratories, we capture sex and age, among other properties.

### CCF Ontology examples

This section presents examples of how the ASCT+B tables, 3D reference organs, landmarks, and experimental tissue block data are represented using CCF Ontology classes and properties. We render ontology class names in mixed case italics (e.g., *Spatial Entity*, where the first letter of each compound word is capitalized), set ontology properties in lower-case italics with underscores between words (e.g., *part_of* or *has_object_reference*), and use bold italics for OWL reserved words (e.g., ***SubClassOf***). We use normal orthography to describe what a class or property represents.

#### HRA reference organs

An excerpt of how a reference organ is represented using the CCF Ontology is shown in Fig. 2. The middle panel details how the Visible Human Project female left kidney (#VHFLeftKidney) reference organ is represented as a *Spatial Entity*. This *Spatial Entity* has well-defined 3D spatial dimensions and a link to a Graphics Language Transmission Format Binary (GLB) file of type *Spatial Object Reference* that captures the 3D shape of this anatomical structure. GLB files are binary forms of the Graphics Language Transmission Format (glTF), a lightweight, open, and extendable file format for 3D assets and scenes, allowing fast loading of 3D content, especially in web browsers. The reference object is properly placed in the female reference body via a *Spatial Placement* that defines 3D scaling, among other properties. Highlighted in orange are *representation_of* and *organ_owner_sex* that are encoded as OWL Annotation Properties (in lower left) with persistent uniform resource locator (PURL) links to Uberon ID 0004538 for “left kidney” in the Biological Structure information﻿ (top right) and to the Logical Observation Identifier Names and Codes (LOINC) ID LA3–6 for “female” sex in the Specimen information (in lower right). Spatial entities representing 53 3D reference organs, 8 extraction sites, and 35 landmark extraction sites for a total of 97 are listed in **Zenodo Table 2**^27^.

#### Experimental tissue data

**Fig. 3:**
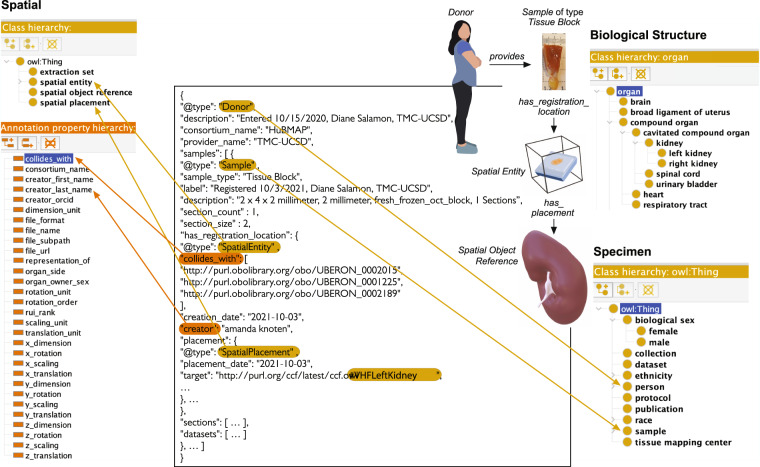
HRA tissue block description. A *Donor* (which *is_a Person*) provides a *Sample* of type *Tissue Block* with properties such as *Biological Sex* and annotation properties such as *Creator* to keep track of provenance. The *Tissue Block* is registered with the Biological Structure using annotation property *collides_with* with three PURL links to UBERON:0002015 (kidney capsule), UBERON:0001225 (cortex of kidney), and UBERON:0002189 (outer cortex of kidney) that all have GLB files with proper *Spatial Placement* in the 3D Reference Object Library.

The CCF Ontology is also used to describe experimental data. Figure 3 exemplifies an instance of a *Sample* where its type is a tissue block that was provided by a *Donor* which is a *Person* in the Specimen class hierarchy (in lower right). The *Sample* instance contains information about the sample registration recording through the *has_registration_location* property and where the data submitter had placed the sample relative to the reference organ through the *has_placement* property. In our example data, we can tell the sample is located in the female left kidney as “#VHFLeftKidney” is mentioned by the *target* property. 3D object collision events are used to compute which anatomical structures are inside of a tissue block (see CCF Ontology Applications section for details). Using annotation property *collides_with* (in lower left), we get PURL links to three anatomical structures—UBERON:0002015 (kidney capsule), UBERON:0001225 (cortex of kidney), and UBERON:0002189 (outer cortex of kidney)—which have corresponding GLB files that define their shape as well as their size, location, and rotation in relationship to the HRA 3D reference system. If anatomical structures have cell types and biomarkers listed in the ASCT+B table, it is possible to generate lists of cell types and biomarkers commonly found in these colliding anatomical structures. Note that the partonomy of anatomical structures described by the ontology makes it possible to retrieve all sub-anatomical structures together with all the cell types found in these.

#### Landmarks and extraction sites

There exist two types of spatial objects that ease tissue registration via the RUI (see Fig. 1 and the CCF Ontology Applications section). Landmarks such as the ‘bisection line’ for the kidney help align tissue blocks with real-world or man-made objects. Predefined tissue extraction sites used in the Genotype-Tissue Expression (GTEx)^28^ project can be assigned to many tissue blocks (e.g., in situations when tissue is always extracted from the very same spatial location in a human donor body^29^, see also the GTEx Standard Operating Procedures Library at https://biospecimens.cancer.gov/resources/sops/library.asp). Both types of spatial objects are represented as a *Spatial Entity* that has been tagged as a member of an *Extraction Set*. Each *Spatial Entity* is associated with a 3D reference organ that has properties such as ‘organ owner sex’ and ‘left/right,’ which uniquely identify the organ. Examples of 364 extraction sites from four consortia are given in **Zenodo Table 3**^27^.

#### ASCT+B partonomy

Among others, ASCT+B tables record relationships among anatomical structures and cell types. These relationships are represented in the CCF Ontology using the annotation properties *ccf_part_of* and *ccf_located_in*. To build the existential restriction expressions for partonomy in the CCF Ontology, we test each ‘anatomical structure to anatomical structure’ and each ‘cell type to anatomical structure’ relationship to see if it corresponds to a valid *SubClassOf* or *part_of (BFO:0000050)* relationship (asserted or inferred) in the source ontologies (Uberon and CL), adding all valid relationships to the CCF Ontology. For example, in the kidney ASCT+B table (v1.2), we find the following relationships:

glomerular mesangial cell *ccf_located_in* glomerular mesangium *ccf_part_of* mesangium *ccf_part_of* renal corpuscle

These all validate as *part_of* relationships (existential restrictions) and so are added to the CCF Ontology as relationships between 1) anatomical structures

*mesangium* ***SubClassOf**** part_of* ***some**** renal corpuscle*

*glomerular mesangium* ***SubClassOf**** part_of* ***some**** mesangium*

and 2) between cell types and an anatomical structures, for example,

*glomerular mesangial cell* ***SubClassOf**** part_of* ***some**** glomerular mesangium*

Note that we intentionally retain the annotation properties alongside with the existential restriction expressions to be able to query the original ASCT+B table structure in the CCF Ontology through SPARQL, a semantic query language for RDF data.

This allows us to safely take advantage of OWL reasoning to query the ontology. For example, we can use the transitivity of *part_of* to infer that all glomerular mesangial cells are part of a renal corpuscle.

#### ASCT+B biomarker set

The ASCT+B tables also record the relationship between a cell type and a set of biomarkers that is commonly used to characterize the cell type. If cell types have biomarkers that characterize them listed in the ASCT+B tables, then the class *Sequence Collection (SO:0001260)* and the properties *has_characterizing_marker_set (RO:0015004)* and *has_marker_component* are used to construct the ontology statement in the CCF Ontology, for example,

*glomerular mesangial cell* ***SubClassOf**** has_characterizing_marker_set* ***some**** (Sequence Collection* ***and***

*(has_marker_component* ***some**** periostin)* ***and***

*(has_marker_component* ***some**** PIEZO2)* ***and***

*(has_marker_component* ***some**** ROBO1)* ***and***

*(has_marker_component* ***some**** ITGA8)* ***and***

*(has_marker_component* ***some**** PDGFRB)* ***and***

*(has_marker_component* ***some**** SYNPO2))*

The interpretation of this statement is that the six cell-type markers *periostin* (HGNC:16953), *PIEZO2* (HGNC:26270), *ROBO1* (HGNC:10249), *ITGA8* (HGNC:6144), *PDGFRB* (HGNC:8804), and *SYNPO2* (HGNC:17732) together are sufficient to distinguish cells of type *glomerular mesangial cell* (CL:1000742) from others found in the same anatomical context (here, the anatomical structure *glomerular mesangium* [UBERON:0002320]). This assertion might be made based on human expertise or on experimental data. An example of the latter is the lung atlas work by Travaglini *et al*.^30^, who found that a minimum of 3–6 differentially expressed gene biomarkers are sufficient to uniquely identify 58 transcriptionally unique cell types in the lung for the spatial locations and demographic groups that were sampled. Note that tissue samples from different spatial locations (e.g., those not yet sampled at single-cell level) or donor populations (older donors or different ethnicities) might have different cell-type populations and characterizing biomarker sets.

### CCF Ontology data specifications

The CCF Ontology provides the vocabularies to enable data exchange across consortia (e.g., see HRA reference organ description example in Fig. 2 or HRA tissue block description in Fig. 3).

**Fig. 4:**
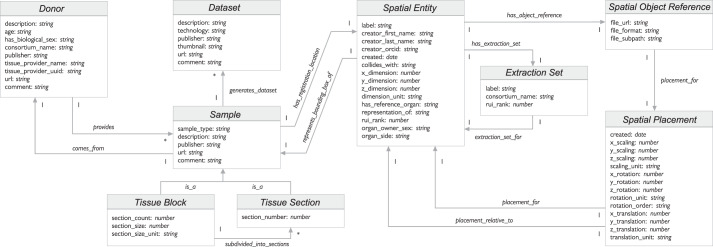
Data specification diagram. Major entities and their relationships are shown as used in JSON-LD. Note that a *Sample comes_from* a *Donor*; it is either a *Tissue Block* or a *Tissue Section*. A *Dataset* might be generated from a *Sample*. A *Sample* typically has a *Spatial Entity* which defines its size in relation to a *Spatial Object Reference* via a *Spatial Placement*.

The data-specification diagram of major CCF Ontology classes and interlinkages is shown in Fig. 4. Some class pairs are linked through inverse relationships (e.g., a *Donor provides Sample*; *Sample comes_from Donor*). Most relationships are of single cardinality (indicated by a 1 on the arrow), whereas some are of multiple cardinality (indicated by an asterisk). Examples of multiple cardinality include: a donor can provide multiple tissue samples, a sample can be used to generate multiple datasets, a tissue block can be subdivided into multiple tissue sections.

The class *Spatial Entity* conceptualizes the notion of 3D objects in the CCF Ontology. The coordinate framework for placing the 3D objects relative to each other is modeled through the class *Spatial Placement*. A 3D reference organ is a *Spatial Object Reference* with a *Spatial Placement* inside of the 3D HRA coordinate system (see example in Fig. 2). A *Tissue Block* is a *Sample* that *has_registration_location* defined via the *Spatial Entity* class relative to a *Spatial Object Reference* (see example in Fig. 3).

### CCF Ontology applications

**Fig. 5:**
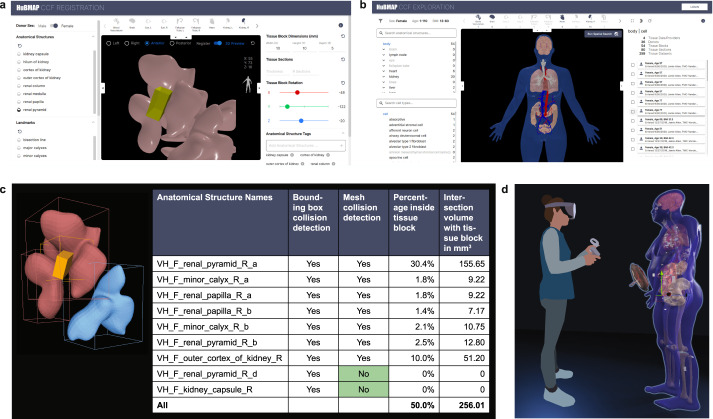
Applications. (**a**) Registration User Interface (RUI) can be used to assign specimen, biological structure, and spatial metadata. (**b**) Exploration User Interface (EUI) supports specimen, biological structure, and spatial search and exploration. (**c**) Mesh-level collision detection improves semantic tagging. (**d**) HRA Organ Gallery with life-size 3D representations of the Visible Human Project dataset.

The CCF.OWL v2.0.1 and the CCF API are used by a variety of applications. Four applications are detailed here and shown in Fig. 5. Each uses interlinked specimen, biological structure, and spatial data to provide unique functionality to users.

#### HuBMAP Portal

The Tissue Registration User Interface (RUI, see Fig. 5a) was integrated into the HuBMAP Ingest Portal (https://ingest.hubmapconsortium.org) to support the HRA-compliant registration of tissue samples^31^. RUI users first pick a 3D reference object that matches the organ from which tissue was extracted; then, they change the 3D size, position, and rotation of a virtual cube (in yellow in Fig. 5a) such that the values match the real-world tissue block properties—in relation to a 3D reference organ. Collision detection (i.e., the intersection of the virtual cube with the reference organ anatomical structures) is used to assign anatomical structure tags (see lower right of RUI). Spatial, biological structure, and specimen information is generated in JSON format. A stand-alone version of the RUI is available (https://hubmapconsortium.github.io/ccf-ui/rui/) and data can be downloaded locally and shared freely as LOD so it becomes available via the CCF-API. The Exploration User Interface (EUI, see Fig. 5b) uses the CCF-API to let users explore RUI-annotated data using specimen, biological structure, and spatial attributes. For example, it is possible to filter tissue data collected by different consortia from different donors using properties such as donor sex and age, cell types, or spatial location. The code for both applications is freely available (https://github.com/hubmapconsortium/ccf-ui). The EUI has been successfully integrated into the GTEx portal (https://gtexportal.org/home/eui) and will be added to the SenNet portal (https://data.sennetconsortium.org); a preview of the EUI on the SenNet portal is here: https://sennetconsortium.github.io/ccf-preview/ccf-eui.html.

#### Mesh-level collision detection

The current RUI performs collision detection at the bounding box level, generating more anatomical structure tags than desirable. Mesh-level collision detection checks if a tissue block collides with the 3D triangular meshes that define the surface of each reference anatomical structure. Figure 5c shows an example of a tissue block (in yellow) colliding with three different renal pyramids in the female right kidney. Using bounding box collision, all three renal pyramids collide. Using mesh-level collision, only the two pink colored pyramids—but not the blue colored renal pyramids—collide. The data table shows Boolean values for collision detection using a traditional minimal bounding box (MBB)-based approach vs. mesh-based collision; differences are highlighted in green. The two columns on the right show the percentage and volume of different anatomical structures inside of the tissue block using mesh-level collision. Note that the total volume inside the tissue block is less than 100%, as there is empty space between anatomical structures in the current 3D Reference Object Library.

To automate mesh-level collision detection, the HRA 3D Reference Objects were reviewed and re-meshed as needed to produce watertight meshes. For example, holes were filled using hole-filling algorithms^32^ and non-manifold meshes were detected automatically but corrected manually by professional medical illustrators using 3D tools such as Zbrush (https://www.maxon.net/en/zbrush) and Maya (https://www.autodesk.com/products/maya/overview). Next, a multi-level spatial index^33^ was computed for each anatomical structure (i.e., the primitives such as faces, vertices, and edges of each mesh), improving query performance by a factor of 10 compared with the traditional filter-refine paradigm used by PostGIS(https://postgis.net/). An in memory on- demand approach was used for data management, and an efficient collision detection algorithm^12,43^ was employed to compute the type and volume of anatomical structures within each tissue block. Last but not least, we computed the volume that is not inside any existing anatomical structures (e.g., that is outside of the organ, possibly indicating that a re-registration via the RUI is needed). All code has been made available on GitHub (https://github.com/hubmapconsortium/ccf-tissue-block-annotation).

### HRA Organ Gallery in VR

The HRA Organ Gallery lets users explore the male and female human reference bodies in a 3D virtual environment using an Oculus Quest 2 virtual reality headset. The VR setup makes it possible to explore the HRA data across different scales—from encountering a whole body in real size (see Fig. 5d), zooming into single organs and studying their many anatomical structures, or examining cell-type populations within specific anatomical structures^11^. The HRA Organ Gallery utilizes the CCF API to retrieve all 53 reference organs published in the third HRA release and 350 HuBMAP tissue blocks publicly available on August 25, 2022 (see **Zenodo Table 3**^27^). While the male and female reference body share many organs (such as brain, kidneys, colon), the male has the prostate, while the female body features the ovaries, fallopian tube, uterus, and placenta. Using HuBMAP IDs associated with each tissue block, additional specimen, provenance, and other data can be retrieved via the HuBMAP Entity API (https://entity.api.hubmapconsortium.org/) and made available via the application available on GitHub (https://github.com/cns-iu/ccf-organ-vr-gallery).

## Discussion

The CCF Ontology v2.0.1 and the CCF API are used both in several production applications across consortia and in research and training. Still, the current ontology model and API code have a number of limitations that have become obvious when using them in different contexts and for different use cases. Some modeling decisions (e.g., representing spatial entities and relationships using 3D meshes registered into a common coordinate system) have proven valuable while other decisions (e.g., representing landmarks as an *Extraction Site*) have been confusing for users and will be revised in future iterations. We discuss key limitations, lessons learned, and next steps here to empower other investigators who work on atlas design or applications that link the HRA to other linked open life sciences data^34^.

### Coverage

The CCF Ontology does not cover all digital objects published in HRA v1.2. The next version of the ontology will aim to capture cell-type typologies, validated Organ Mapping Antibody Panels (OMAPs)^35^, 2D reference objects for micro-anatomical structures such as functional tissue units (FTU)^36^, and information on supporting scholarly publications and experimental data. We will model organs that have branching relationships with anatomical structures (e.g., blood vasculature, lymph vasculature, and peripheral nervous system) differently from those that do not (e.g., kidney or heart). We will add and expand the definitions of ontology classes and properties. In the current CCF Ontology, all original entities and relationships from the ASCT+B tables are preserved (as *ccf* property triples) as annotation property relationships (i.e., they can be queried separately from relationships derived from Uberon/CL or other reference ontologies).

### Improved validation

Currently, biological structure is validated against Uberon and CL. Going forward, we also will validate biomarkers against HGNC and UniProt. References to entities that do not exist on relevant ontologies will be added to issue trackers for eventual inclusion into existing ontologies. For example, since the publication of HRA v1.2, 127 terms from the provisional CL have been included and are now properly validating, and all 127 were published in HRA v1.3. Note that the current set of 26 ASCT+B tables features links to LMHA for lung cell types and Provisional CL (PCL) for brain cell types; future revisions of the HRA will aim to replace them by links to CL.

### Data provenance

Novel experimental data can substantially change the HRA reference and mapping procedure. Hence, it is of utmost importance to capture the provenance of all data and code used in atlas creation and to recompile the HRA when new evidence becomes available. Going forward, we expect to see multiple biomarker sets (e.g., generated using different cell-type annotation tools) per cell type population; these can be encoded using Boolean AND between all required biomarkers from one set and OR between biomarker sets; this will likely lead to ontology reasoner scaling issues. Future versions of the CCF Ontology will support complete provenance chains for all digital objects and code using the W3C PROV ontology (https://www.w3.org/TR/prov-overview/)^37^.

### Spatial accuracy

To understand the limitations of tissue registration using the Registration User Interface (RUI), we performed a user study with 42 subjects^31^. Results show that RUI users can perform tissue block matching tasks at an average of 5.88 degrees rotation accuracy and 1.32 mm position accuracy (at an average of 22.6 seconds per task after 8.3 tasks in a series of 30 identical tasks). Going forward, we will implement spatial registration using biomolecular data that is only now becoming available for some anatomical structures. Over time, with sufficient high-quality data from thousands of human donors, the HRA will become more stable and data registration more accurate.

### Proper separation between ontologies and data instances

The CCF Ontology v3.0 will model HRA-relevant vocabularies, application ontologies, and references using the LinkML (https://linkml.io/) general purpose modeling language. The HRA will continue to use terms from the CCF Ontology to detail all specimen-related, structural biology, and spatial data. Experimental data from various consortia will be modeled using LinkML and advertised as 5-star Linked Open Data (https://www.w3.org/2011/gld/wiki/5_Star_Linked_Data). That is, the CCF Ontology defines the vocabularies shared by HRA and experimental data; LinkML is used for the data specification. Data instances will not be part of the CCF Ontology, but they will be made available via a separate data management system. Concretely, data (e.g., extraction sites) and instances (data about tissue blocks) will be stored in a HRA Knowledge Base. The 2/3D reference object files will be stored in multiple places, including GitHub repositories and content delivery network (CDN) services. The CCF API already has code to access the HuBMAP Data Warehouse via the Search API and to advertise relevant HuBMAP data as LOD that is compatible with HRA.

### Novel use cases

A series of qualitatively new use cases will be developed to showcase the value of combining HRA with other LOD data—e.g, from the Gene-Centric Common Fund Data Ecosystem (CFDE) Knowledge Graph (https://maayanlab.cloud/gene-kg/downloads), the STRING - Protein-Protein Interaction Networks Functional Enrichment Analysis database (https://string-db.org/), and the Scalable Precision Medicine Oriented Knowledge Engine (SPOKE) graph database (https://cns-iu.github.io/spoke-vis/home), which federates more than 30 open datasets into a public data commons of health-relevant knowledge^13^.

### Scaling up

Efficient data management, spatial queries and algorithm implementations are needed to scale up to the 37 trillion cells that make up the human body^2^ and to truly capture human diversity. CCF data is a core component that will be accessed very frequently by many database queries. To guarantee fast, real-time query response of CCF data, we will continue to monitor and improve the implementation through techniques such as in-memory data management, hybrid relational and graph-based storage, and parallel processing.

## Methods

### CCF Ontology source data

The third HRA (v1.2) was authored by 154 unique experts. The experts were selected based on their demonstrated expertise in human anatomy and biomolecular data; monthly working group meetings were used to resolve questions and report progress; all new and revised digital objects were presented and discussed in the working group before they were submitted, reviewed, and published. The 3D reference organs in this HRA were modeled using textbooks such as Gray’s *Anatomy*^38^, Netter’s *Atlas of Human Anatomy*^39^ and similar sources that are cited in the 3D model metadata. In total, 456 publications with Digital Object Identifiers (DOIs) are listed as scholarly evidence in the third HRA. Experimental data generated by four consortia (HuBMAP, SenNet, KPMP, and GTEx) were mapped onto the HRA; in December 2022 there exist 1,161 spatially registered tissue blocks that link to more than 6,000 biomolecular datasets.

### CCF Ontology editing, building, and release

A new release of the HRA is published every six months. The CCF Ontology derived from the HRA data is published as a CCF.OWL graph database and associated CCF API shortly thereafter. Experimental datasets compliant with earlier versions of the HRA might need to be re-registered (e.g., if ASCT+B table terminology or 3D reference organs changed).

The “Standard Operating Procedure (SOP) Document for Ontology Editor” (https://github.com/hubmapconsortium/ccf-ontology/blob/7170116226871adf62cd47e65fccae0ec8d3671c/src/ontology/README.md) details how (1) to download and setup the development environment using GitHub, (2) to add new reference organs to the CCF Ontology, (3) to update an existing reference organ in the CCF Ontology, (4) to build the CCF Ontology, and (5) to release the CCF Ontology.

**Fig. 6:**
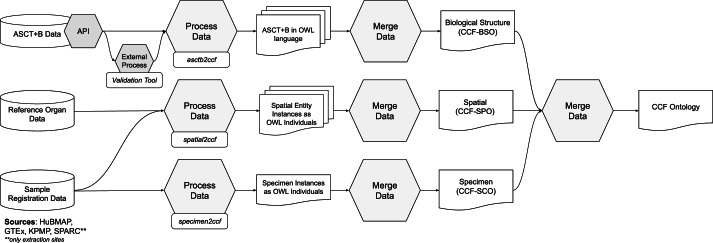
CCF Ontology Generation Pipeline. ASCT+B tables are validated and semantically enriched using the Validation Tool; in parallel, 3D Reference Organ data and crosswalk to ASCT+B tables are processed to compute the HRA. Sample Registration Data tissue blocks with RUI locations and anatomical structure tags can be retrieved for HRA construction or during HRA usage.

The general CCF Ontology generation pipeline is shown in Fig. 6. The ASCT+B tables (in top left) are read by a *Validation Tool* (https://github.com/hubmapconsortium/ccf-validation-tools) that compares whether anatomical structure and cell type terms are valid and if these relationships are true according to Uberon and CL/PCL using the UberGraph^40^ SPARQL endpoint. All terms and relationships that already exist in Uberon and CL/PCL are added to the Biological Structure information﻿. When a term or relationship does not validate, the closest alternative match to another term in the ASCT+B Table is found and added. If no match is found, then ontology change request tickets (GitHub issues) are generated for missing anatomical structures, cell types, and relations for inclusion in existing ontologies. The result is a “partonomy” that reports *part_of* relationships between each pair of terms (anatomical structures to anatomical structures; cell types and anatomical structures—called *located_in* in the ASCT+B tables. A second pipeline uses the ROBOT tool^41^ to read the ASCT+B tables, augment them with information from reference ontologies (e.g., it adds metadata such as synonyms, definitions, or class hierarchies), and adds this additional data to the Biological Structure information﻿. Both processes generate weekly reports that are used to improve ASCT+B tables and to generate ontology change requests. Next, the *asctb2ccf* (https://github.com/hubmapconsortium/asctb2ccf) tool reads the validation tool report together with the original relationships from the ASCT+B table and generates a characterizing biomarker set for each cell type. *Spatial Object Reference* data (in middle left) is read by the *spatial2ccf* (https://github.com/hubmapconsortium/spatial2ccf) tool that generates instances of *Spatial Entity*, *Spatial Object Reference*, and *Spatial Placement* classes defined by the CCF Spatial Ontology. *Sample* registration data (in lower left) from HuBMAP, SPARC, KPMP, GTEx is available as LOD (see **Zenodo Table 3**^27^ for examples). Each *Extraction Set* has a well-defined *Spatial Placement* (e.g., using the Registration User Interface in the CCF Ontology Applications section). Multiple tissue blocks can be associated with the same *Extraction Set* (e.g., GTEx has 29 *Extraction Sets* for more than 5,000 datasets). The *asctb2ccf* tool reads the anatomical structure tags and generates Biological Structure information﻿ data (e.g., predictions of cell types commonly found in these anatomical structures). The *spatial2ccf* tool reads spatial data and generates Spatial information data. The *specimen2ccf* (https://github.com/hubmapconsortium/specimen2ccf) reads *Donor, Tissue Block*, and *Sample* data and generates Specimen information data.

The CCF-Slim Ontology (ccf-slim.owl) captures HRA data exclusively (no experimental data), and it is used in the Registration User Interface (see CCF Ontology Applications section).

### CCF Ontology validation

As discussed, existing ontologies do not (yet) completely capture healthy human anatomical structures, the cell types located in them, and the biomarkers that are typically used to characterize these cell types. We used a python-based *Validation Tool* (https://github.com/hubmapconsortium/ccf-validation-tools) to compare whether anatomical structure and cell type terms are valid and if these relationships are true according to Uberon and CL/PCL using UberGraph^40^. When comparing the ASCT+B tables on June 13, 2022 with Uberon (May 17, 2022 version) and Cell Ontology (CL, Feb 16, 2022 version), there were 1,620 terms that do not validate; 1,290 of these are from blood vasculature and peripheral nervous system (PNS). In both cases, expert mappings are mostly to FMA, which has a more detailed representation of human vasculature and peripheral nervous system anatomy than Uberon has. We are in the process of adding missing terms and relationships to Uberon.

Many of the *part_of* relationships between anatomical structures validate, but only a minority of the anatomical structures to cell type relationships validate. This is, in-large part, because of the use of very generic cell types (e.g., fibroblast, T-cell) in specific locations in the ASCT+B tables. In some cases, these indicate the need for new cell type terms, specific to the location; in other cases (e.g., non-resident immune cells), improved semantic representations may be needed.

Recent technological advances in single-cell analysis make it possible to classify cell types based on their transcriptomic profiles and to derive sets of marker transcripts that are sufficient to identify these cell types. This has resulted in a large number of new cell types that are not yet recorded in CL but are captured in the ASCT+B tables. For example, there are 953 ASCT+B table cell type entries covered by HRA (v1.2) that are not mapped to a CL ID. Some of these terms will be mapped in future releases, whereas others represent cell types not yet known to CL. We are using provisional cell-type terms and submitting new term request tickets so new terms can be added to CL.

As for biomarkers, the 3rd HRA (v1.2) features 2,842 total with 1,959 of type gene, 878 proteins, 3 proteoforms (in eye), 1 lipid (in placenta), and 1 metabolite (in eye). Of these, 447 biomarkers have provisional ontology IDs. Other gene and protein biomarkers are linked to HGNC.

Technical validation of the CCF Ontology was performed by implementing diverse value-added services; see section CCF Ontology Applications. We are in the process of developing a framework for specifying and testing queries against the CCF Ontology based on competency questions. Each competency question will specify a query and a minimal answer (i.e., some set of terms that the query should return, based on the intention of ASCT+B table author) together with an endpoint and a formal query in the correct syntax for the endpoint. The specified queries can then be run systematically against the endpoints, reporting success, or not. Test queries might include: “All cells in location X of type Y”, “All cells in location X of type Y expressing marker Z.”, “Location(s) of cell type X”, or “Location(s) of cell type X expressing marker Z”.

## Data Availability

The CCF.OWL v2.0.1 is available through the NCBI BioPortal Ontology Browser (https://bioportal.bioontology.org/ontologies/CCF). It is findable via https://purl.org/ccf/releases/2.1.0/ccf.owl. A GitHub repository with basic information about working with the ontology can be found at https://github.com/hubmapconsortium/ccf-ontology. N3.js (https://www.npmjs.com/package/n3) is used as a graph database. There are 266,576 triples in the graph database that holds the content of the CCF.OWL v2.0.1. The size of all 53 3D reference objects (GLB files) in HRA v1.2 is 172 MB. CCF.OWL v2.0.1 represents major digital objects of the HRA v1.2, including 26 ASCT+B tables, 54 3D reference objects, and a crosswalk that associates anatomical structures in both. In addition, the data covers landmark organ sets which are used in the tissue registration user interface to facilitate tissue block placement in 3D organ models; these were not published with DOIs, but do appear in the CCF Ontology. CCF Ontology classes and properties are listed in **Zenodo Table 1**. Spatial entities representing 53 3D reference organs, 8 extraction sites, and 35 landmark extraction sites for a total of 97 are listed in **Zenodo Table 2**. Experimental tissue data comprises 364 tissue blocks or registration sites and links to more than 6,000 datasets, see **Zenodo Table 3**^27^. Weekly run term and relationship validation reports are available at https://github.com/hubmapconsortium/ccf-validation-tools/tree/master/reports. Documentation and standard operating procedures have been published via the HRA Portal, https://hubmapconsortium.github.io/ccf. The CCF API supports programmatic access to CCF.OWL v2.0.1 data; for exemplary queries see the companion website at https://cns-iu.github.io/ccf.owl-paper-supporting-information. The CCF.OWL v2.0.1 data and API are used in the HuBMAP and GTEx portals, the Registration and the Exploration User Interfaces, 3D mesh-level collision detection, and the HRA Organ Gallery.

## Code availability

All data and standard operating procedures are released under Creative Commons Attribution 4.0 International (CC BY 4.0). All code was released under the MIT License and can be accessed in GitHub at https://github.com/hubmapconsortium/ccf-ontology.

ASCT+B APIs:

API Endpoint (includes interactive documentation): https://mmpyikxkcp.us-east-2.awsapprunner.com

API Documentation: https://hubmapconsortium.github.io/ccf-asct-reporter/docs/api

OpenAPI specification: https://mmpyikxkcp.us-east-2.awsapprunner.com/asctb-api-spec.yaml

CCF-API:

API Endpoint (includes interactive documentation): https://ccf-api.hubmapconsortium.org

API Documentation and OpenAPI specification: https://ccf-api.hubmapconsortium.org

API Database backend is N3.js: https://github.com/rdfjs/N3.js

Code to instantiate/use CCF Database: https://github.com/hubmapconsortium/ccf-ui/tree/main/projects/ccf-database

CCF-User Interfaces:https://github.com/hubmapconsortium/ccf-ui

Validation Tools:https://github.com/hubmapconsortium/ccf-validation-tools
